# Visualization of the Sorption of Nickel within Exopolymer Microdomains of Bacterial Microcolonies Using Confocal and Scanning Electron Microscopy

**DOI:** 10.1264/jsme2.ME18134

**Published:** 2019-02-23

**Authors:** John R. Lawrence, George D.W. Swerhone, Thomas R. Neu

**Affiliations:** 1 Environment and Climate Change Canada 11 Innovation Blvd. Saskatoon, Saskatchewan Canada, S7N 3H5; 2 Department of River Ecology, Helmholtz Centre for Environmental Research—UFZ Brueckstrasse 3A 39114 Magdeburg Germany

**Keywords:** Newport Green, SEM, microdomains, exopolymer, confocal

## Abstract

The sorption and distribution of nickel, a common metal contaminant in aquatic systems, were assessed in bacterial microcolonies using a combination of fluorescent staining with Newport Green and confocal laser scanning microscopy (CLSM) with confirmation by scanning electron microscopy (SEM) and X-ray microprobe analyses. CLSM with Newport Green, selected fluor-conjugated lectins, and DNA staining allowed for the discrimination of the microdomains present in the microcolony exopolymeric matrix and detection of bound nickel. This approach avoided the artefacts associated with drying and fixation required by analytical electron microscopy. The results obtained indicated that specific microcolonies within river biofilms sorbed nickel within limited microdomains present in the complex tripartite exopolymeric matrix surrounding bacterial cells. Sorption occurred such that nickel was concentrated within the exopolymeric matrix, but not directly associated with cells. These microdomains appeared to have neutral pH and be dominated by negatively charged residues favoring the sorption of nickel and other cations. These results also suggest an important role for specific community members in the sorption and concentration of metals in aquatic biofilm communities.

Microorganisms are capable of interacting with and sorbing metals, even in relatively dilute solutions. Bacterial cells frequently act as mediators for the enrichment of various metals. Trudinger (31) noted that microorganisms may directly concentrate, change physicochemical conditions, generate organic matter, or catalyze reactions that result in the concentration of metals. Gadd (8) also reported that microbial cells and microcolonies may impact metal sorption processes by altering local pH and Eh, and through the production of organic and inorganic nutrients as well as metabolites. The phenomenon of the hindered penetration of metals into biofilms and flocs has also been reported. Rose and Cushing (26) demonstrated the hindered penetration of metals in periphyton with radiotracers, suggesting that this plays a significant role in microbial resistance to metals, particularly in biofilm communities.

The surfaces of bacteria are highly interactive with the external environment. Numerous studies have demonstrated the involvement of bacterial cell walls, which are charged at neutral pH, in the formation of fine-grained minerals (3, 6, 28). The role of external exopolysaccharides (EPS), including lipopolysaccharides, in metal sorption and mineral formation has been shown (4, 15, 21). Lalonde *et al*. (14) reported the adsorption of metals onto surface organic functional groups, including carboxylic and amino acids associated with cells, extracellular structures, and EPS. Differences in these surface organic groups and variations in environmental parameters, including pH, temperature, nutrients, electron acceptors, and specific metal species, influence the magnitude of sorption (22, 25). The river epilithion hosts a wide variety of microorganisms and mineral precipitation has frequently been reported in river biofilms (20). Mixed species biofilms may sequester metals at concentrations of 4 to 5 orders of magnitude above that in the surrounding waters (27). Friese *et al*. (7) showed increases in K, Ca, Cr, and Mn that were up to 100- to 60,000-fold higher in the biofilm matrix than in the bulk water for Elbe river biofilms. Similarly, Lunsdorf *et al*. (20) demonstrated the sorption of Al, Fe, and Mn to the exopolymer of microcolonies in Elbe river biofilms. Thus, the exopolymer matrix of microcolonies and biofilms represent a critical sink in terms of understanding the partitioning and accumulation of metals in aquatic environments. It has also become clear that the nature of exopolymer microdomains (18) and the microenvironment (19) at the microcolony scale are complex. These factors may all have a significant influence on the migration of toxic heavy metals in both industrial and natural settings. This is particularly relevant given the ubiquity of biofilms on all surfaces in aquatic environments, such as rivers. In the present study, we utilized confocal laser scanning microscopy (CLSM), metal-sensitive fluorescent probes, fluor-conjugated lectins, nucleic acid probes, scanning electron microscopy (SEM), and X-ray microprobe analyses to characterize specific microcolonies within river biofilms that are capable of sequestering metals, such as nickel.

## Materials and Methods

### Biofilm cultivation

Microbial biofilms were cultivated in a rotating annular bioreactor (RAB) with natural river water as the inoculum and sole source of carbon and nutrients (16, 24). The reactor contained removable polycarbonate strips that allowed for the examination of biofilm materials at the desired intervals. Biofilms were exposed to the following concentrations of nickel for 24 h prior to microscopic examinations: 0, 1, 500, 1,000, and 10,000 μg L^−1^. Samples were rinsed 3 times with filtered (0.2 μm) and sterilized river water prior to CLSM and SEM analyses.

### CLSM

A Bio-Rad MRC-1024 confocal laser scanning system, equipped with a krypton-argon laser and mounted on a Nikon Microphot-SA microscope (Nikon Canada, Mississauga, Ontario, Canada), was used to non-destructively obtain images of biofilms. Observations of RAB strips were made with the following water immersible lenses: 63×, 0.9 numerical aperture (NA) (Zeiss, Jena, Germany) and 40×, 0.55 NA (Nikon). Microcolonies were readily identified based on their characteristic morphologies and lectin-binding properties. The colonies studied were frequently observed and occurred at a density of >1×10^5^ cm^−2^ of biofilm (18). Therefore, we were able to select and examine numerous colonies and study >10 examples of this colony type in detail.

### SEM

Samples of biofilm material were air-dried and carbon-coated before being subjected to examination by SEM. Samples were observed using a JEOL Model 660 system (JEOL USA, Peabody, MA, USA) at an accelerating voltage of 20 kV and current of 10 nA in the backscatter mode. The chemical composition of the biofilm material was elucidated by wavelength dispersive spectrometry using a JEOL Model 660 SuperProbe. The quantitative electron microanalysis was performed with settings of 15 kV, 10 nA, and 1.5-μm spot size. Acquisition times were 40–60 s per element and correction factors were applied in accordance with Bence and Albee (1).

### Staining procedures

**Fig. 1:**
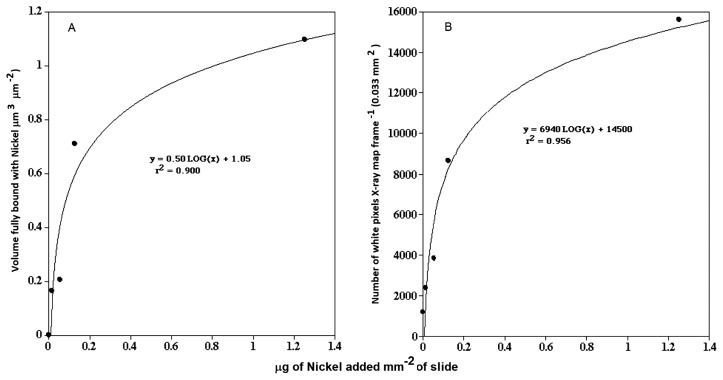
Graphs showing the relationship between the concentration of nickel in the bulk phase and the fluorescent intensity of Newport Green in the exopolymer of microcolonies (A) and nickel levels detected by X-ray mapping (B).

CLSM biofilm analyses involved single, double, and triple labeling procedures, *e.g*. green (excitation [ex] 488, emission [em] 522/32), red (ex 568, em 605/32), and far red (ex=647, em 680/32). A panel of fluorescein isothiocyanate (FITC), tetramethyl rhodamine isothiocyanate (TRITC), and CY5-conjugated lectins allowed the distribution of glycoconjugate-binding sites to be visualized in biofilm materials, including *Triticum vulgaris*-TRITC and *Tetragonolobus purpureaus-*CY5 (23). The nucleic acid stain SYTO 9 (Thermo Fisher Scientific, Waltham, MA, USA) acted as a general stain for all bacterial cells. In addition, the metal-sensitive probe Newport Green (Thermo Fisher Scientific), which increases its fluorescent signal in the presence of metals, such as nickel and cadmium, was used to provide an indication of metal distribution in fully hydrated microcolony and biofilm materials. Biofilms on 1×1 cm^2^ coupons cut from 1×10 cm polycarbonate reactor slides were exposed to 100 μL of the selected Ni solutions for 1 h. They were subsequently rinsed three times with sterile river water (0.2-μm filter) before staining. A 2.4 mM Newport Green (molecular weight: 801.638) solution was prepared in DMSO and 100 μL of the solution was added to a surface of 1 cm^2^ of the biofilm on a polycarbonate coupon and incubated in the dark at room temperature for 15 min prior to observations with CLSM with excitation/emission wavelengths corresponding to *ca*. 505/535 nm. Biofilm material exposed to 0, 1, 500, 1,000, and 10,000 μg L^−1^ Ni was used to create a standard curve of nickel concentrations in the liquid phase versus the fluorescence intensity of Newport Green (average grey value, Fig. 1A). Parallel subsamples were prepared for X-ray mapping and the assessment of nickel levels in biofilm and microcolony materials (Fig. 1B).

### Image analyses and 3D-reconstruction procedures

Digital image analyses, using NIH Image v 1.63, of CLSM optical thin sections in each of the 3 channels were used to assess average grey levels. In addition, three color red-green-blue projections of the biofilms and microcolonies were computed.

## Results and Discussion

As reported by Tourney and Ngwenya (30), few studies have examined the sorption of metals in naturally occurring biofilms; however, a number of outcomes have been described, including an inability to clarify the importance of various mechanisms (14). These findings suggest that Ni associated with pre-existing Mn and Fe minerals found in microbial sheaths, instead of cells and EPS (11). We herein attempted to utilize a combination of observations of dried samples and SEM as well as fully hydrated biofilm samples utilizing fluorescent probes and CLSM to observe the sorption of nickel to a biofilm as well as a specific microcolony type common in our rotating annular reactor biofilms cultivated with South Saskatchewan River water (16). We previously employed fluorescent lectin binding analyses (FLBA) and FLBA-FISH analyses to describe the morphology of a distinctive, abundant microcolony type (>1×10^5^ cm^−2^ of biofilm) growing in river biofilms (18). The application of a series of fluorescent oligonucleotide probes identified the bacterium as a member of *β*-*Proteobacteria*. Further FLBA and fluorescent probe analyses revealed that a complex EPS arrangement consisted of the following: (i) a region surrounding each individual cell similar to the bacterial capsule, (ii) additional distinct intercellular EPS external to the first layer and filling between cells, and (iii) a boundary layer of EPS that was external to all colony members. Other studies noted the existence of similar highly ordered EPS arrangements in microcolonies using electron microscopy (13, 20). Additional *in situ* CLSM microscopy studies of this microcolony subsequently revealed the structural, microenvironmental, and metabolic implications of this complex EPS structure (19). That study described the presence of microdomains associated with phosphatase enzyme activity, a filter cascade of decreasing pore sizes from the external surface to the cell surface, and significant gradients of pH—from the cell interior (pH 7) to the microcolony interior (pH 4+) with a gradient of increasing pH (pH 7+) to the colony exterior (19). We speculated that the existence of these microdomains within the microcolony may have implications for other phenomena, including the sorption of metals and other contaminants. It is clear that a critical aspect of EPS is its highly reactive nature. Previous studies clearly demonstrated that the sorption of contaminants, including metals, occurs within biofilms and may be associated with particular bacteria and exopolymeric substances (20, 21). Combinations of electron microscopy, spectromicroscopy, and destructive mass spectrometric confirmations utilizing dried and fixed biofilm materials have been employed. However, to understand the nature of the interactions of metals and other contaminants with exopolymeric substances, it is critical to conserve their 3D structures and highly hydrated nature (10, 33, 34). Wolfaardt *et al*. (33) utilized CLSM and fluorescence and showed that specific biofilm regions and EPS residues were associated with the sorption of the herbicide diclofop methyl. Lawrence *et al*. (17) characterized river biofilms using fluor-conjugated lectins to define EPS and fluorescent immunochemical techniques to localize the herbicide atrazine on microcolonies within the biofilm matrix. The metal-sensitive fluorescent reporter Newport Green is a complexing agent that fluoresces upon binding with nickel, zinc, or cobalt, and, thus, may be useful for the visualization of metals, including nickel, zinc, and cobalt, in solution as well as in gels and biofilms, as reported previously (34). Wuertz *et al*. (34) showed that Newport Green complexed nickel in gelatin and agarose and successfully applied it in fully hydrated biofilms and flocs to detect nickel outside cells using CLSM. However, Hao *et al*. (9) noted that although Newport Green was responsive to metals, it exhibited poor selectivity and did not produce unambiguous findings. Although this is limiting, it is clear that the confirmation of fluorescence reporters for the presence of metals must always be reached using SEM, TEM, or even scanning transmission x-ray microscopy (STXM) (9–11, 32, 34, 35).

**Fig. 2:**
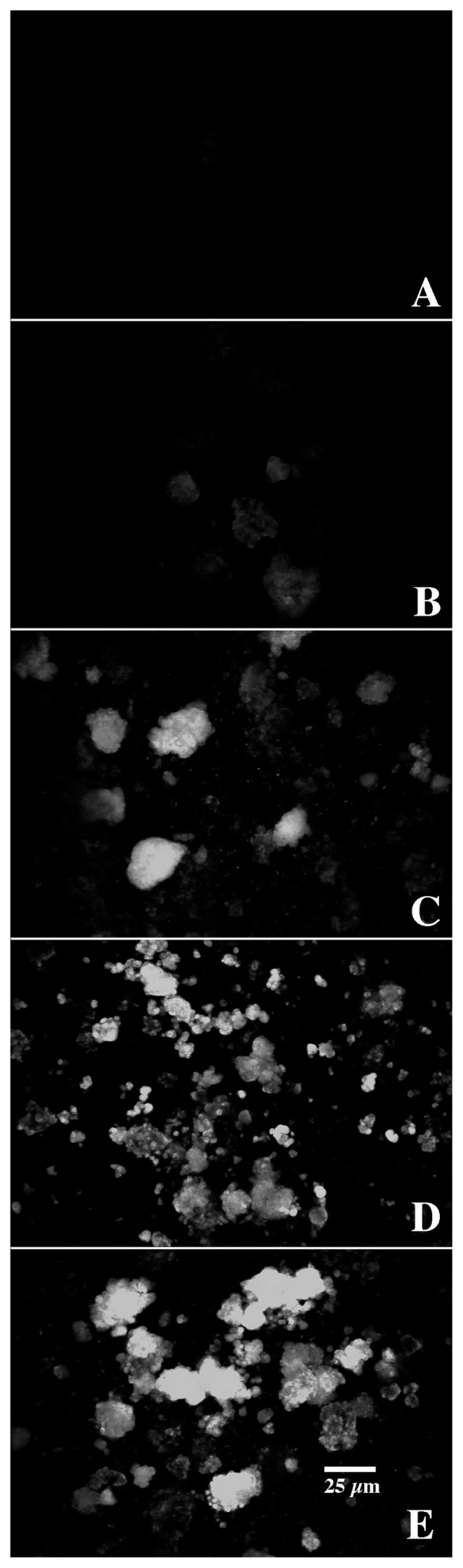
CSLM fluorescence images showing the distribution and intensity of Newport Green fluorescence in (A–E) control=0, 1, 500, 1,000, and 10,000 μg L^−1^ nickel-treated river microcolonies. Images show the increasing concentration of Ni in the biofilm and microcolonies with higher levels of Ni in the bulk phase.

**Fig. 3:**
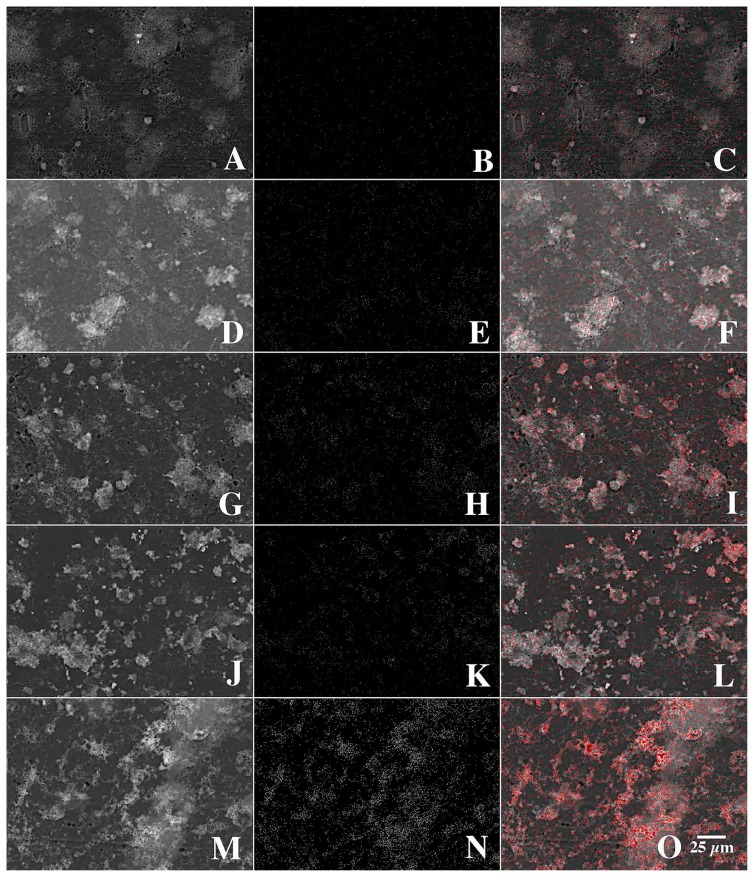
Back scatter (left column A–M), X-ray mapping (center column B–N), and overlay (right column C–O) (red pixels indicate the presence of nickel) images of biofilms treated with 0, (A, B, C) 100, (D, E, F), 500, (G, H, I), 1,000, (K, L, M), and 10,000, (M, N, O) μg L^−1^ nickel. Images show the increasing concentration of Ni in the biofilm and microcolonies (red pixels) with higher levels of Ni in the bulk phase, paralleling the results obtained with CLSM fluorescence imaging.

**Fig. 4:**
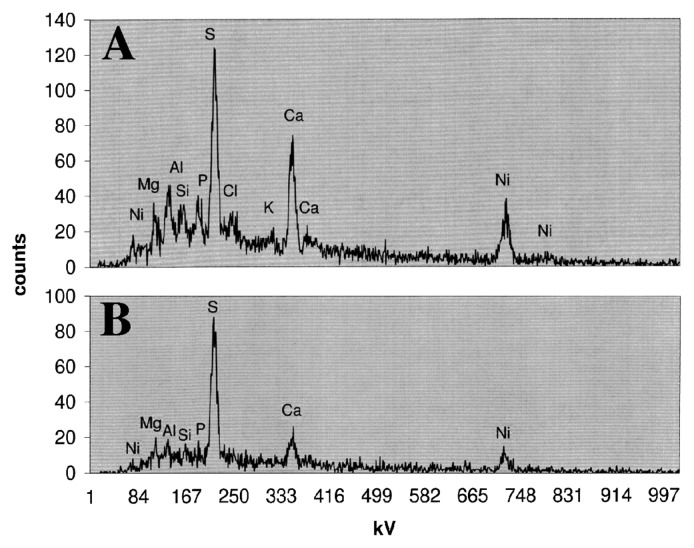
X-ray microprobe scans of microcolonies (A) and general biofilms excluding microcolonies (B) treated with 10,000 μg L^−1^ nickel. The detection minimum for this analysis was 2,000 μg L^−1^ w/w in dried biofilm material. Since peak heights were proportional to concentrations, the microcolony material had approximately 4-fold higher levels of nickel than general biofilms.

In keeping with this approach, in addition to detection using Newport Green and CLSM, we mapped the presence of nickel in biofilms and microcolonies in parallel studies using SEM and quantitative electron microanalyses. CLSM micrographs of biofilms exposed to control=0, 1, 500, 1,000, and 10,000 μg L^−1^ nickel are shown in Fig. 2A–E and revealed stronger fluorescence with increasing nickel exposure. X-ray mapping of these samples at a similar magnification (Fig. 3A–O) showed a similar pattern of stronger signals with increasing nickel exposure. The results of these analyses confirmed the presence of nickel associated with the biofilm and specific microcolonies (Fig. 4A and B). These results also showed a relationship between the concentration of nickel used in exposure and the increasing intensity of Newport Green fluorescence in the biofilm (Fig. 2A–E). Furthermore, the concentration of nickel detected using CLSM imaging corresponded to changes in nickel levels detected by X-ray mapping (Fig. 1B and 3A–O). Finally, X-ray microprobe scans (Fig. 4A and B) confirmed that nickel was present in both the biofilm in general and microcolonies specifically, indicating that microcolonies have approximately 4-fold the amount of nickel detected in the general biofilm excluding microcolonies. These results support the efficacy of Newport Green in this particular environment and confirm the presence of nickel and its preferential sorption to *β*-proteobacterial colonies, as confirmed by fluorescent *in situ* hybridization analyses (18, 19).

**Fig. 5:**
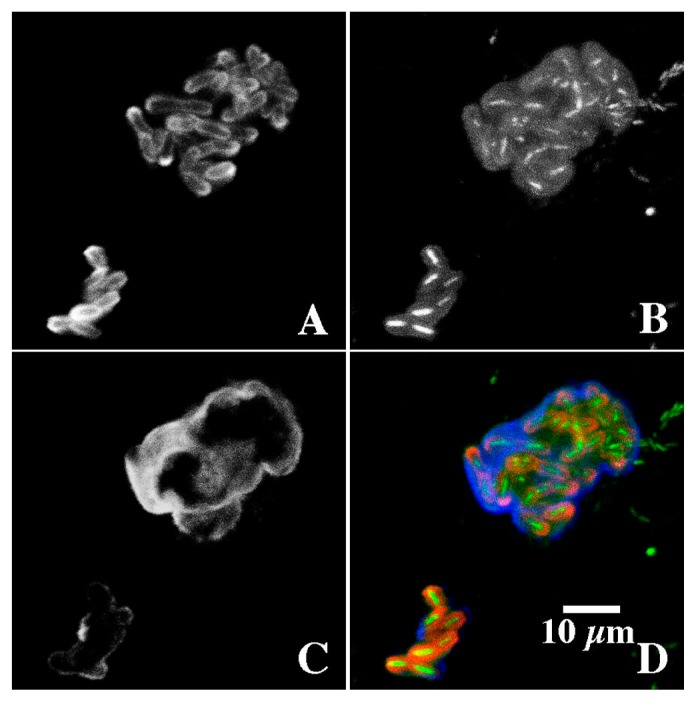
CLSM images of the application of fluor-conjugated lectins (A) *Triticum vulgaris*-TRITC, (B) SYTO 9, and (C) *Tetragonolobus purpureaus-*CY5. Results indicated that 3 types of binding sites were associated with the exopolymer matrix and these corresponded to three structural regions: (i) the cell surface, (ii) capsular material around individual cells, and (iii) a third layer encompassing the entire colony. This outer layer and the boundary with the capsular region appeared to be involved in the binding of Ni (see Fig. 7).

**Fig. 6:**
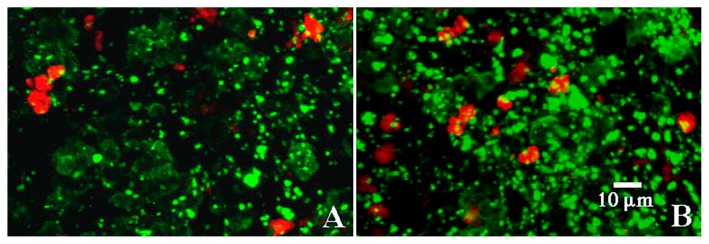
Images of biofilms stained with (A) TRITC-conjugated *Triticum vulgaris* lectin and a FITC-conjugated anionic 70k mw dextran, and (B) FITC-conjugated polyanionic 70k mw dextran. The limited yellow overlay indicates that the microcolony did not bind negatively charged dextrans.

**Fig. 7:**
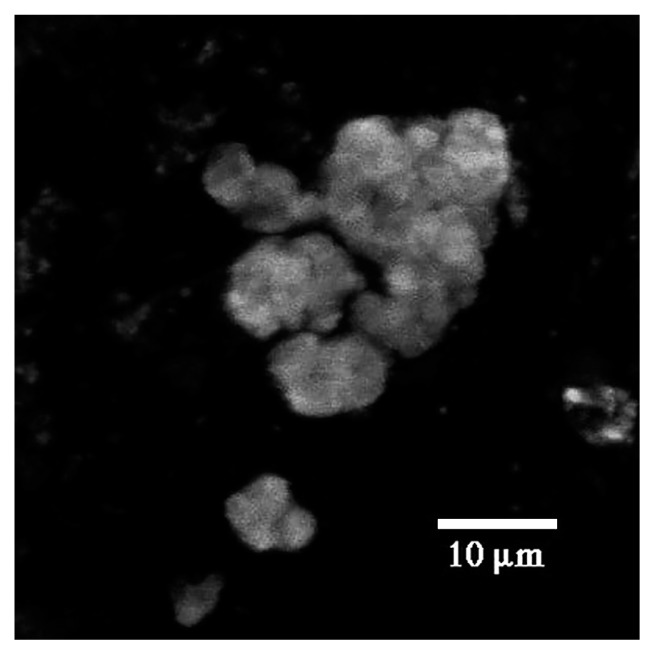
CLSM image of a microcolony after the sorption of nickel and staining with Newport Green. Nickel binding appeared to be associated with the outer regions and the boundary with the capsular region of the microcolony. Note: These observations also parallel those made in 2D with low magnification CLSM (Fig. 2) and SEM (Fig. 3) showing the entire outline of the microcolony enriched with nickel.

The four-fold increase observed in nickel sorption by the microcolonies and the nature of complex microdomains present in the microcolonies suggested that higher resolution imaging using CLSM and a suite of fluorescent probes, including Newport Green, are beneficial. Bacterial microcolonies frequently exhibit a complex structured exopolymeric matrix with several components creating classic microdomains (5). As reported by Lawrence *et al*. (18) who examined a number of bacterial microcolonies, there are a broad range of glycoconjugates present in EPS. These include cell-associated layers with galactose, glucose, mannose, N-acetyl glucosamine, and glycoproteins, intercellular layers with detectable residues of fucose, mannose, and glucose, and an outer shell potentially containing fucose, galactose, N-acetyl glucosamine and N-acetyl glucosamine oligomers. In the present study, the application of the fluor-conjugated lectins *T. vulgaris*-TRITC (N-acetylglucosamine residues and oligomers) and *T. purpureaus-*CY5 (fucose), along with the DNA stain SYTO 9, confirmed that at least three types of binding sites were associated with the exopolymer matrix. These binding sites corresponded to three structural regions: (i) the cell surface, (ii) capsular material around individual cells, and (iii) a third layer encompassing the entire colony, which is consistent with previous findings (18, 19). Fig. 5 shows individual channels and a three-color reconstruction of the microcolony, revealing the cellular and exopolymer domains present in the microcolony under examination. The patterns shown in Fig. 5 are consistent with the existence of chemical-physical microdomains surrounding the individual bacterial cells within the microcolony. Hoffman and Decho (12) suggested that this represented an efficient matrix for the localization of extracellular enzymes and their hydrolytic products. Lawrence *et al*. (19) confirmed that enzyme activities, such as alkaline phosphatase and glucose oxidase, were active within specific regions of the EPS matrix. Furthermore, they demonstrated the existence of pH gradients in these colonies, which may be of significance for the sorption of contaminants. Beveridge (2) and others (6) noted that the EPS molecules of most bacterial species were negatively charged under circumneutral pH conditions due to the presence of predominantly carboxylic and phosphoryl functional groups. In accordance with previous findings, the application of a FITC-conjugated polyanionic 70K mw dextran to biofilms revealed a lack of binding to the microcolony, which is consistent with a low density of positively charged functional groups (Fig. 6). However, since lectin binding may be based in part on charge interactions (23), *T. vulgaris* lectin binding may interfere with the binding of the anionic dextran. Neu *et al*. (23) also noted that the addition of lectins may result in the masking of specific regions of mono-, di-, and oligosaccharides in the matrix and/or potentially create new targets that are recognized by subsequent lectins. For example, when *T. vulgaris*-lectin was added in the absence of *T. purpureas*-lectin, binding to the external-EPS layer was observed (Fig. 6). Stumm and Morgan (29) also showed that reactions between metallic ions and polymeric substances were strongly influenced by pH. The sorption of metallic ions is enhanced at neutral pH due to increases in these ionized acidic groups. We noted steep gradients of pH within microcolonies, with the outer boundary of EPS exhibiting pH of *ca*. 7 (19). This is consistent with a pH microdomain favoring the sorption of metals in this region of the microcolony. In accordance with these results, the application of Newport Green, as shown in Fig. 7, suggested that nickel sorption was predominantly associated with the outer region of the microcolony and the boundary with the capsular region, consistent with the existence of physical chemical microdomains favoring the partitioning of metals to the exterior of the microcolony. These results are also in agreement with the appearance of nickel sorption in low magnification CLSM (Fig. 2) and SEM images (Fig. 3) showing the outline of the microcolony enriched with nickel. This phenomenon may also favor the mitigating negative effects of metals that may occur at the cell interface, as noted by Ueshima *et al*. (32). This study speculated that EPS function to minimize the toxic effects of Cd due to EPS binding of toxic aqueous Cd. Similar effects may occur in natural microcolonies due to this partitioning of metals and reductions in their activity in relation to the bacterial cell. EPS have been suggested to absorb various quantities of a range of metals and the relative importance of the cell wall and EPS in metal binding has not yet been established (32). Our results indicate a significant role for EPS in the current case.

## Conclusions

The present results suggest that the binding of Ni occurred within the matrix of river biofilms and was associated with the presence of a specific morphologically distinct microcolony type. The results of lectin-binding analyses revealed a complex exopolymeric matrix with multiple regions of differing chemistries. These results indicate that fluorescent probes, such as Newport Green, are useful for detecting metals within fully hydrated biofilm materials. The results obtained with Newport Green were consistent with those of X-ray mapping. The results of X-ray microprobe analyses suggest that 4-fold more nickel was concentrated in microcolonies than in general biofilms. The present study supports the importance of the bacterial community structure and presence of specific organisms for assessing the partitioning and accumulation of toxic elements, such as nickel, in river biofilm communities.
